# High-fidelity simulation programs in ICU-related ethical non-technical skills training: A narrative review

**DOI:** 10.62838/jccm-2026-0002

**Published:** 2026-04-30

**Authors:** Alexandra-Maria Boldis, Cristina Petrisor, Darius Turcas, George-Calin Dindelegan

**Affiliations:** Iuliu Hațieganu University of Medicine and Pharmacy, Cluj-Napoca, Romania

**Keywords:** high-fidelity simulation programs, trainees, ethical considerations, non-technical skills

## Abstract

**Objective:**

Is there a place for non-technical skills training in the ICU? And what teaching strategy should we implement in this process? This narrative review analyzes the benefits of teaching ethics in the ICU environment by applying high-fidelity simulation scenarios to real-life situations, thereby improving communication, moral reasoning, self-reliance, cooperation, and perceptual skills.

**Methods:**

In the literature, there are few publications on the training of ICU residents in non-technical skills and ethical dilemmas using high-fidelity simulations. After searching and scoping the database, we have identified 8 publications relevant to this narrative review.

**Results:**

In the reviewed studies, the main topics discussed and rehearsed using simulations were as follows: communicating an adverse event during anesthesia in one study, delivering bad news in two studies, the ethics of end-of-life care, and the do-not-resuscitate order in three studies, and ethical non-technical skills such as communications, teamwork and confidence in emergent real-life situations in four studies.

**Conclusions:**

Developing a more structured approach to teaching ethics-related events is important, particularly in critical care settings. All reviewed studies reached the same conclusion: high-fidelity simulation training is an educational strategy for ICU residents to develop a foundation in ethical considerations and moral reasoning by improving ethical non-technical skills, such as confidence, communication, teamwork, delivering bad news, and end-of-life care.

## Introduction

In Intensive Care Units (ICUs), medical and auxiliary personnel frequently encounter complex ethical dilemmas that require moral reasoning and rational decision-making [1,2]. Educational strategies are diverse, and choosing the right one depends on evidence-based medicine. Throughout the teaching process, traditional methods are not always suitable for achieving this goal, and a more innovative approach is necessary [3, 4]. Therefore, the implementation of high-fidelity simulation programs in ICU-related ethical dilemmas training is supported by current literature as an applicable tool in teaching ethics [4,5]. Moreover, the simulation of real-life ethical dilemmas encountered in the ICU is intended to support the trainee’s future decision-making and problem-solving, with a focus on key concepts such as altruism, autonomy, human dignity, integrity, and social justice [6,7,8].

In the high-stakes environment of Intensive Care Units, a fast and precise reaction time is crucial. During the residency program, trainees develop technical skills through learning, practice, and work to ensure safe practice in their profession. Regarding non-technical skills, assimilation is significantly more challenging and requires special attention because it relies heavily on human judgment. When faced with ethical dilemmas, residents are more likely to make errors due to deficiencies in reasoning, limited experience, and cognitive load that cannot be readily accessed or processed [9]. Therefore, there should be growing interest in the cognitive factors underlying medical decisions, complementing technical skills and enhancing the quality and safety of care [10]. By practicing moral reasoning and fast and slow thinking when faced with an ethical dilemma, the healthcare professional can enhance individual and collective situational awareness, effective and safe communication, and establish safer methods for crisis management, leadership, and teamwork [11,12,13].

## Methods

We searched the PubMed database from October 2023 to June 2025. We used the following keywords: non-technical skills, ethics education, moral reasoning, ICU trainees, ICU residents, ICU fellows, high-fidelity simulation, medical ethics training, simulation-based learning, and high-fidelity simulation programs in the ICU. We found very few publications related to the training of ICU residents in non-technical skills and ethical dilemmas using high-fidelity simulations [5,14,15,16,17,18,19,20]. There are studies concerning simulation-based teaching in critical care, anaesthesia, and emergency medicine, and the participants in these studies are nurses, residents, and physicians [10,21,22,23,24,25,26,27,28,29,30,31,32,33,34,35,36]. Regarding the relationship between simulation and ethical care in the intensive care unit, all papers analysed for this narrative review used simulation-based training to help learners improve their ethical decision-making processes during diagnosing and treating patients [37,38].

## Results

**Fig. 1.**
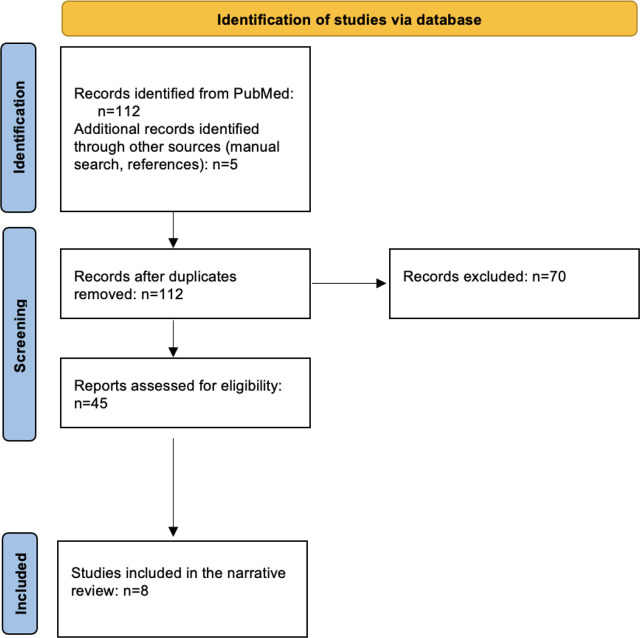
Study flow chart

During our online search of the database, we found a few articles that referred to teaching ethics using high-fidelity simulation programs, but only a limited number (8) addressed ICU trainees. The study inclusion process for this narrative review is shown in Figure 1. A more detailed search strategy is presented in Appendix.

The studies included in this narrative review are heterogeneous; participants include ICU trainees, but also nurses, physicians, respiratory therapists, and administrative staff. The main ethical, non-technical skills discussed and rehearsed vary across the included publications. All of the studies focus on different forms of communication, for example, delivering bad news in two studies[14,18], communicating an adverse event during anesthesia in one study[5], the ethics of end-of-life care, and the do-not-resuscitate order in three studies [5,18,19], and teamwork and confidence in emergent real-life situations in four studies [15,16,17,20]. Five of these studies focused on ethical dilemmas [5,14,17,18,19], and the other three on improving communication and self-confidence among ICU residents through simulations [15,16,20]. Depending on the principle developed and practiced during the simulations, the main outcome patterns vary between the studies: from improved communication comfort among the critical care trainees [14,18], to improved teamwork, role appointment [15,16,17], solving different ethical issues [5], and managing the relinquishment of life-sustaining treatment [5,18,19].

Another important aspect of the selected publications is the characteristics of interventions. All studies employed the principle of high-fidelity simulation, using realistic clinical scenarios that enabled ICU trainees to practice complex skills. By contrast, intervention durations vary across studies, ranging from 3 months (the shortest) [16] to 10 years (the longest) for a high-fidelity simulation-based educational strategy [5].

It is difficult to draw objective conclusions from the studies included in this review, partly because of the heterogeneity in sample sizes and publication quality. There are only three interventional studies with participant counts above 50 [15,17,20]; the others have small sample sizes, with 10–37 enrolled trainees and medical staff [5,14,16,18,19]. From this perspective, the results obtained after each simulation-based training session include subjective endpoints, such as self-reported comfort, and rarely evaluate real-world outcomes. Therefore, the reproducibility of these studies is low, and the risk of bias is high (Table 1).

**Table 1.** Study’s description, participants, and characteristics of the interventions

**Author (Year)**	**Country**	**Design**	**Sample Size**	**Intervention**	**Duration**	**Description**
Tanoubi et al. (2020)	Canada	short report	10 cohorts of residents (24 residents in each cohort)	High-fidelity simulation	10 years	3 simulation scenarios involving circumstances with different ethical issues (instruction in critical incident disclosure, communication regarding patient awareness under general anesthesia, and physicians’ perspectives on overriding do-not-resuscitate orders in the context of iatrogenic cardiac arrest)
Ghoneim et al (2018)	USA	interventional study	15 participants (NICU trainees)	A simulation-based training intervention focusing on the SPIKES protocol for breaking bad news	1 year	Participant delivery of bad news to a standardized parent (actor in the role of a parent).
Colman et al (2019)	USA	mixed methodological observation cohort study	165 participants (nurses, critical care fellow, respiratory therapist)	Implementation of team training through simulation	21-month period	The simulation program comprised 30 mandatory workshops, each lasting 3 hours and featuring three scenarios based on real-life ICU cases, using mannequin settings, trained embedded participants, and facilitator prompts. After the simulations, there was an improvement in communication, teamwork, and role appointment.
Figueroa et al (2012)	USA	interventional study	37 participants	The course included: didactics and high-fidelity simulation-based training	3 months	The implementation of Team STEPPS protocol and its incorporation into both, the course content and simulation scenarios, resulted in improved communication within the multidisciplinary PICU team.
Downar et al (2012)	Canada	interventional study	51 trainees	This communication workshop incorporated a short didactic session and four simulated family meetings, using trained professionals as standardized family members.	5 years	Findings of the workshop included ethical and legal knowledge and communication comfort (before and after the workshop), as well as communication skills. Participation in the practical course significantly enhanced ethical and legal knowledge and improved communication comfort among the critical care trainees.
Arnold et al (2015)	USA	interventional study	38 Pulmonary and critical care fellows	The intervention was a 3-day communication skills workshop incorporating short didactic sessions, faculty skill demonstrations, and practice with simulated families.	2 years	Training focused on three core domains: delivering bad news, achieving consensus on therapeutic goals, and discussing limitations of life-sustaining treatments. Participant self-assessments of competence in 11 key communication points were collected before and after the workshop using a 5-point Likert scale.
Hope et al (2015)	USA	interventional study	31 critical care fellows	A didactic curriculum containing lectures and case discussions on end-of-life care, communication, palliative care, and bioethics was created, supplemented by two simulated family meetings	3 years	Residents participated in 101 family meeting simulation. After following a month-long curriculum, more than 90% of trainees declared increased comfort – either ‘slightly’ or ‘much’ more comfortable – with debates regarding the relinquishment of life-sustaining treatment.
Sung et al (2025)	Taiwan	Prospective interventional study	237 participants, including medical trainees, nurses, respiratory therapists, and administrative staff.	The intervention incorporated two high-fidelity scenarios simulating real emergencies.	5 years	The group performing was evaluated using the Team Emergency Assessment Measure (TEAM). Methodical questionnaires granted qualitative feedback that was analysed thematically. Involvement in the program allowed an improved communication, teamwork and collaborative skills between healthcare professionals working in vulnerable environments.

## Discussions

When we discuss communication training for end-of-life decisions, palliative care, delivering bad news, discussing prognosis, and explaining critical care interventions for high-risk patients, it is challenging to establish a standardized communication pattern due to population heterogeneity, variable study information, and the broad scope of clinical interventions [19,39].

Critical care often places physicians in situations that require balancing patient safety with the performance of complex, invasive procedures, necessitating strong communication skills to achieve optimal outcomes [40]. The importance of high-quality care in the ICU depends upon clinicians’ correspondence with critically ill patients and their surrogates, and proactive communication strategies have been associated with improvements in patient- and family-centered outcomes [41,42]. Learning communications skills from senior colleagues is often insufficient, and simulation methodologies must be followed efficiently to improve the teaching of non-technical skills to medical trainees [42,43,44].

It has been shown that healthcare simulations are relevant to critical care and can be used to optimize the quality of care for intensive care patients [45]. There is no doubt that workshop training is valuable for technical skills; simulation-based communication training improves guidance for patients and surrogates during critical illness [46,47,48].

Therefore, the ICU environment requires more structured educational programs, such as high-fidelity simulation, to develop and implement a new curriculum for teaching ethical principles [5,10,41,49].

In the literature, we find a few examples of teaching ethics through high-fidelity simulation; Tanoubi’s study describes an innovative model. They intended to introduce the key ethical considerations and to implement them through scenario-based rehearsal for anesthesiology residents. The same idea is found in the study by Krimshtein et al., which involved ICU nurses as participants and documented improvements in non-technical skills, such as communication [26]. Using high-fidelity simulation scenarios, they’ve created an immersive experience that reflects real-life events and ethical dilemmas and guides trainees and auxiliary personnel through moral reasoning, ultimately contributing to the acquisition of essential non-technical skills [5,26]. The same idea of clinical reasoning is developed among medical students in Mutter’s interventional study, by using this case-based teaching technique [34].

Over the years, the process of delivering bad news to patients’ families has been the subject of numerous studies aimed at improving communication skills. The ICU environment, particularly the Neonatal Intensive Care Unit (NICU), is frequently discussed in the literature because clinicians must manage complex situations and require proper training to develop the non-technical skills necessary to resolve them [50]. The development of Ethical Life Support provides evidence of the importance of teaching medical ethics to critical care and emergency clinicians, with the new acronyms: A-Acknowledge, B-Be aware, C-Communicate, D-Deal [51].

Simulation-based training to improve communication skills is particularly relevant given the high risk of death among patients in ICU wards [52]. Today, the modern ICU environment has changed, and the burden of delivering patient-focused care rests heavily on healthcare providers [1]. Families’ and patients’ expectations are consistently high, and doctors must be taught essential non-technical skills for communication and interprofessional education to improve clinical decision-making [53,54]. This is clearly demonstrated in Tzu-Ching Sung’s paper, where the inclusion of high-fidelity simulation training in a prospective mixed-methods study led to improved performance in managing high-risk scenarios encountered in everyday practice (in emergency and critical care departments) [20]. The effectiveness of teamwork and communication is also illustrated in the study by Garbee et al., who used a high-fidelity simulation scenario with students and anesthesia nurses. Despite the study’s small sample size, this new teaching modality improved participants’ non-technical skills [23]. The publication by Cerra et al. is another example supporting the importance of high-fidelity simulation-based learning in enhancing self-confidence, communication, satisfaction, and self-efficacy [29].

In contrast, clinical skills training platforms are used more frequently. Using this type of high-fidelity patient training resulted in improvements in medical knowledge, medical behaviour, and care quality [54,55]. With high-fidelity patient situation simulation, used for teaching ethics and non-technical skills, critical residents and students can achieve important non-technical communication objectives [5,10,56,57,58]. However, these high-fidelity situation simulations are time-consuming and can be expensive if employed with advanced technological support, but they are highly effective in improving the quality of education and raising confidence [6,7,59–60].

This narrative review’s limitations stem from subjectivity, the small sample sizes of the included interventional studies, reliance on self-reported improvements, and the short-term nature of the outcomes.

## Conclusion

There is no certain evidence that using high-fidelity simulation ethics training has a positive clinical impact, but all the participants in all of the studies, trainees, nurses, and students, have given satisfaction surveys and good oral comments. Using this teaching method leads to improvements in ethical, non-technical skills, such as communication and teamwork, self-confidence, delivering bad news, and addressing the longstanding dilemma of end-of-life care[5,10].

Regarding the present and the future, all ethical dilemmas encountered in the ICU will be amplified by the global burden and individual concerns, and all healthcare professionals should benefit from proper training to address any ethical encounter, keeping in mind the Hippocratic Oath: “*First, do no harm*.”[61].

## Appendix

**Table:**

**Database**	**Search equations**	**Filters**
PubMed (Medline)	“non-technical skills “OR” ethics education “AND” simulation training”	-
